# Penta­carbonyl-1κ^2^ *C*,2κ^3^ *C*-(μ-pyrazine-2,3-dithiol­ato-1:2κ^4^ *S*,*S*′:*S*,*S*′)(trimethyl­phosphane-1κ*P*)diiron(I)(*Fe*—*Fe*)

**DOI:** 10.1107/S1600536811046770

**Published:** 2011-11-12

**Authors:** Shang Gao, Chun-Ai An, Qian Duan, Da-Yong Jiang

**Affiliations:** a School of Materials Science and Engineering, Changchun University of Science and Technology, 7989 Weixing Road, Changchun 130022, People’s Republic of China

## Abstract

In the title compound, [Fe_2_(C_4_H_2_N_2_S_2_)(C_3_H_9_P)(CO)_5_], the Fe_2_S_2_ core adopts a butterfly conformation. The PMe_3_ ligand is coordinated in the basal position, roughly *cis* to the Fe—Fe bond. The Fe—Fe distance of 2.4970 (6) Å is relatively short compared to those (*ca* 2.53 Å) found in another monosubstituted diiron compound. A rigid planar dithiol­ate bridge is featured, with an angle of 2.78 (1)° between the Fe—Fe bond and the normal to the pyrazine-2,3-dithiol­ate plane.

## Related literature

The title compound was prepared as a biomimetic model of the [FeFe]-hydrogenase active site. For general background to hydrogenases and iron–sulfur–carbonyl complexes, see: Cammack (1999 ▶); Evans & Pickett (2003 ▶); Liu & Xiao (2011 ▶); Song *et al.* (2005 ▶); Yin *et al.* (2011 ▶). For related structures, see: Li *et al.* (2005 ▶); Liu & Yin (2010 ▶). For the synthesis, see: Durgaprasad *et al.* (2011 ▶).
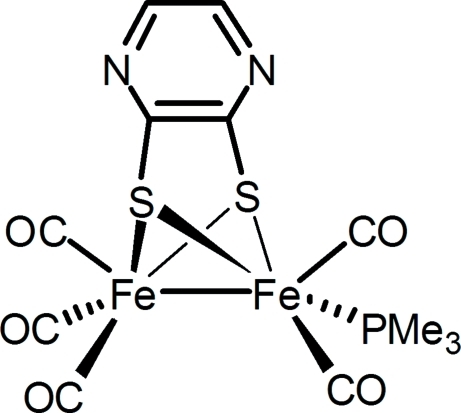


## Experimental

### 

#### Crystal data

[Fe_2_(C_4_H_2_N_2_S_2_)(C_3_H_9_P)(CO)_5_]
*M* *_r_* = 470.02
Orthorhombic, 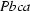

*a* = 14.8307 (2) Å
*b* = 12.1463 (2) Å
*c* = 19.8806 (3) Å
*V* = 3581.25 (9) Å^3^
*Z* = 8
Mo *K*α radiation
μ = 1.97 mm^−1^
*T* = 273 K
0.10 × 0.10 × 0.10 mm

#### Data collection

Bruker APEX CCD diffractometer
Absorption correction: multi-scan (*SADABS*; Sheldrick, 1996 ▶) *T*_min_ = 0.828, *T*_max_ = 0.828
19251 measured reflections
3327 independent reflections
2445 reflections with *I* > 2σ(*I*)
*R*_int_ = 0.064

#### Refinement

*R*[*F* ^2^ > 2σ(*F* ^2^)] = 0.034
*wR*(*F* ^2^) = 0.070
*S* = 1.01
3327 reflections
217 parameters
H-atom parameters constrained
Δρ_max_ = 0.27 e Å^−3^
Δρ_min_ = −0.32 e Å^−3^

### 

Data collection: *SMART* (Bruker, 2007 ▶); cell refinement: *SAINT* (Bruker, 2007 ▶); data reduction: *SAINT*; program(s) used to solve structure: *SHELXS97* (Sheldrick, 2008 ▶); program(s) used to refine structure: *SHELXL97* (Sheldrick, 2008 ▶); molecular graphics: *SHELXTL* (Sheldrick, 2008 ▶); software used to prepare material for publication: *SHELXTL*.

## Supplementary Material

Crystal structure: contains datablock(s) global, I. DOI: 10.1107/S1600536811046770/hy2484sup1.cif

Structure factors: contains datablock(s) I. DOI: 10.1107/S1600536811046770/hy2484Isup2.hkl

Additional supplementary materials:  crystallographic information; 3D view; checkCIF report

## Figures and Tables

**Table 1:** Selected bond lengths (Å)

Fe1—C1	1.803 (4)
Fe1—C2	1.784 (4)
Fe1—C3	1.780 (4)
Fe1—S1	2.2906 (9)
Fe1—S2	2.2893 (9)
Fe2—C4	1.761 (4)
Fe2—C5	1.771 (4)
Fe2—S1	2.2859 (9)
Fe2—S2	2.2783 (9)
Fe2—P1	2.2450 (9)

**Fig. 1.**
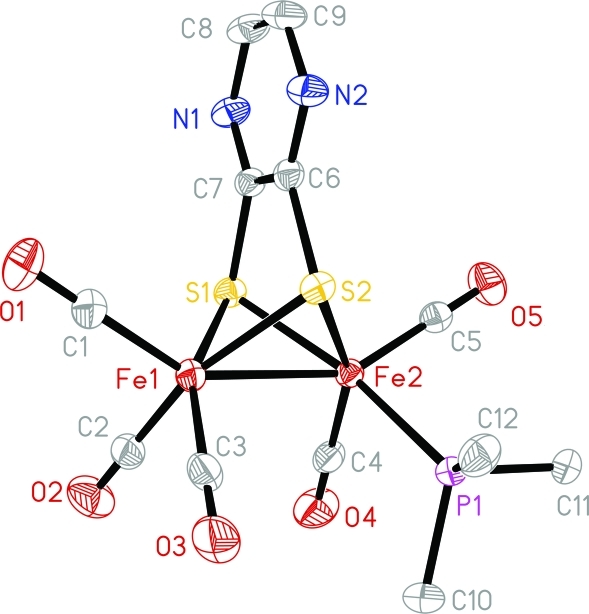
The molecular structure of the title compound, with displacement ellipsoids drawn at the 30% probability level.

## supplementary crystallographic information

### Comment

Hydrogen evolution and uptake in nature is mostly catalyzed by the metalloenzymes called hydrogenases (Evans & Pickett, 2003). Among all types, the [FeFe]-hydrogenases ([FeFe]Hases) are most efficient (Cammack, 1999). The resemblance in structures between the [FeFe]Hase active site and the well known iron-sulfur-carbonyl complexes (Liu & Xiao, 2011; Song *et al.*, 2005; Yin *et al.*, 2011) draws intensive attention to the chemistry of such complexes. The title compound was prepared to mimic structurally the active site of [FeFe]Hases. Herein we report its crystal structure.

In agreement with other reported diiron complexes (Liu & Yin, 2010), the Fe_2_S_2_ unit in the title compound is in a butterfly conformation and each iron atom is coordinated with a pseudo-square-pyramidal geometry (Fig. 1). The deviation of Fe1 atom from the 2S2C-formed basal plane is 0.362 Å. The Fe—Fe distance of 2.4970 (6) Å in the title compound (Table 1) is enlarged by *ca* 0.03 Å than that found in [Fe_2_(µ-C_4_H_2_H_2_)(CO)_6_] (Durgaprasad *et al.*, 2011), indicating the strong electron-donating ability of the PMe_3_ ligand. The Fe—P distance of 2.2450 (9) Å is in good agreement with those in the phosphane-coordinated diiron compounds (Li *et al.*, 2005). The rigid dithiolate bridge is a special feature for the title compound. The calculated plane of the –SC_4_H_2_N_2_S– bridge is nearly a bisect plane of the molecule. The angle between the Fe—Fe bond and the normal of the pyrazine-2,3-dithiolate plane is 2.78 (1)°.

### Experimental

All reactions and operations related to the title compound were carried out under a dry, prepurified nitrogen atmosphere with standard Schlenk techniques. All solvents were dried and distilled prior to use according to standard methods. Me_3_NO and trimethylphosphane were available commercially and used without further purification. The starting material [Fe_2_(µ-C_4_H_2_N_2_)(CO)_6_] was prepared according to the literature procedure (Durgaprasad *et al.*, 2011). [Fe_2_(µ-C_4_H_2_N_2_)(CO)_6_] (0.42 g, 1.0 mmol) and trimethylphosphane (0.08 g, 1.0 mmol) were reacted in CH_3_CN (20 ml) in the presence of Me_3_NO for 20 min at room temperature. The solvent was allowed to evaporate on a rotary evaporator to give a dark-red solid. The crude product was purified by column chromatography on Al_2_O_3_ using CH_2_Cl_2_/hexane as eluent to give a red solid (yield: 0.04 g, 10%). Recrystallization in a CH_2_Cl_2_/pentane solution afforded crystals of the title compound suitable for X-ray study.

### Refinement

H atoms were positioned geometrically and refined as riding atoms, with C—H = 0.93 (CH) and 0.96 (CH_3_) Å and with *U*_iso_(H) = 1.2(1.5 for methyl)*U*_eq_(C).

### Crystal data

**Table:**

[Fe_2_(C_4_H_2_N_2_S_2_)(C_3_H_9_P)(CO)_5_]	*F*(000) = 1888
*M**_r_* = 470.02	*D*_x_ = 1.743 Mg m^−^^3^
Orthorhombic, *P**b**c**a*	Mo *K*α radiation, λ = 0.71073 Å
Hall symbol: -P 2ac 2ab	Cell parameters from 2383 reflections
*a* = 14.8307 (2) Å	θ = 2.4–21.5°
*b* = 12.1463 (2) Å	µ = 1.97 mm^−^^1^
*c* = 19.8806 (3) Å	*T* = 273 K
*V* = 3581.25 (9) Å^3^	Block, red
*Z* = 8	0.10 × 0.10 × 0.10 mm

### Data collection

**Table:**

Bruker APEX CCD diffractometer	3327 independent reflections
Radiation source: fine-focus sealed tube	2445 reflections with *I* > 2σ(*I*)
graphite	*R*_int_ = 0.064
φ and ω scans	θ_max_ = 25.5°, θ_min_ = 2.1°
Absorption correction: multi-scan (*SADABS*; Sheldrick, 1996)	*h* = −17→17
*T*_min_ = 0.828, *T*_max_ = 0.828	*k* = −12→14
19251 measured reflections	*l* = −24→23

### Refinement

**Table:**

Refinement on *F*^2^	0 restraints
Least-squares matrix: full	H-atom parameters constrained
*R*[*F*^2^ > 2σ(*F*^2^)] = 0.034	*w* = 1/[σ^2^(*F*_o_^2^) + (0.0259*P*)^2^ + 0.3172*P*] where *P* = (*F*_o_^2^ + 2*F*_c_^2^)/3
*wR*(*F*^2^) = 0.070	(Δ/σ)_max_ < 0.001
*S* = 1.01	Δρ_max_ = 0.27 e Å^−^^3^
3327 reflections	Δρ_min_ = −0.32 e Å^−^^3^
217 parameters	

### Fractional atomic coordinates and isotropic or equivalent isotropic displacement parameters (Å^2^)

**Table:**

	*x*	*y*	*z*	*U*_iso_*/*U*_eq_	
Fe2	0.03732 (3)	0.27229 (3)	0.07013 (2)	0.03067 (13)	
Fe1	0.01883 (3)	0.18401 (4)	0.18272 (2)	0.03633 (14)	
S2	0.01055 (5)	0.37018 (7)	0.16583 (4)	0.0357 (2)	
S1	−0.09264 (5)	0.18655 (6)	0.10298 (4)	0.0365 (2)	
P1	0.17667 (5)	0.34273 (7)	0.06048 (4)	0.0347 (2)	
C11	0.1950 (2)	0.4175 (3)	−0.01705 (17)	0.0489 (9)	
H11A	0.2555	0.4453	−0.0180	0.073*	
H11B	0.1532	0.4778	−0.0197	0.073*	
H11C	0.1858	0.3690	−0.0546	0.073*	
C6	−0.10919 (19)	0.3860 (3)	0.16280 (15)	0.0343 (7)	
N2	−0.14764 (17)	0.4745 (2)	0.18771 (14)	0.0444 (7)	
C5	−0.0049 (2)	0.3498 (3)	0.00154 (18)	0.0383 (8)	
N1	−0.24488 (17)	0.3034 (2)	0.12409 (14)	0.0457 (7)	
C10	0.2693 (2)	0.2451 (3)	0.05956 (19)	0.0543 (10)	
H10A	0.3253	0.2841	0.0553	0.081*	
H10B	0.2623	0.1958	0.0222	0.081*	
H10C	0.2693	0.2038	0.1007	0.081*	
C7	−0.1565 (2)	0.3014 (3)	0.13199 (15)	0.0353 (8)	
C1	−0.0420 (2)	0.1752 (3)	0.2611 (2)	0.0528 (10)	
C3	0.1313 (2)	0.1954 (3)	0.21316 (18)	0.0466 (9)	
C4	0.0725 (2)	0.1547 (3)	0.02547 (18)	0.0447 (9)	
C9	−0.2385 (2)	0.4767 (3)	0.18021 (18)	0.0507 (10)	
H9A	−0.2703	0.5367	0.1970	0.061*	
C12	0.2126 (2)	0.4416 (3)	0.12345 (18)	0.0530 (10)	
H12A	0.2729	0.4654	0.1137	0.080*	
H12B	0.2111	0.4078	0.1671	0.080*	
H12C	0.1728	0.5039	0.1229	0.080*	
C2	0.0344 (2)	0.0406 (3)	0.16750 (17)	0.0478 (9)	
C8	−0.2849 (2)	0.3953 (3)	0.14932 (19)	0.0522 (10)	
H8A	−0.3471	0.4025	0.1451	0.063*	
O5	−0.03314 (16)	0.3954 (2)	−0.04409 (13)	0.0617 (7)	
O3	0.20369 (17)	0.2019 (2)	0.23081 (15)	0.0719 (9)	
O4	0.09513 (17)	0.0780 (2)	−0.00373 (15)	0.0741 (9)	
O2	0.04365 (18)	−0.0509 (2)	0.15663 (15)	0.0756 (9)	
O1	−0.0802 (2)	0.1710 (3)	0.31047 (16)	0.0905 (10)	

### Atomic displacement parameters (Å^2^)

**Table:**

	*U*^11^	*U*^22^	*U*^33^	*U*^12^	*U*^13^	*U*^23^
Fe2	0.0309 (2)	0.0308 (3)	0.0303 (3)	−0.0002 (2)	0.00212 (19)	−0.0006 (2)
Fe1	0.0361 (3)	0.0381 (3)	0.0348 (3)	0.0003 (2)	0.0004 (2)	0.0057 (2)
S2	0.0333 (4)	0.0371 (5)	0.0366 (5)	−0.0017 (4)	0.0008 (3)	−0.0055 (4)
S1	0.0319 (4)	0.0358 (5)	0.0419 (5)	−0.0025 (4)	0.0002 (4)	−0.0044 (4)
P1	0.0294 (4)	0.0340 (5)	0.0406 (5)	0.0023 (4)	0.0032 (4)	0.0025 (4)
C11	0.042 (2)	0.048 (2)	0.057 (3)	−0.0001 (17)	0.0125 (17)	0.0151 (18)
C6	0.0349 (17)	0.0404 (19)	0.0275 (18)	0.0021 (16)	0.0011 (14)	0.0025 (15)
N2	0.0447 (17)	0.0452 (18)	0.0433 (18)	0.0106 (14)	−0.0005 (13)	−0.0074 (14)
C5	0.0347 (18)	0.040 (2)	0.040 (2)	−0.0022 (15)	−0.0006 (16)	−0.0049 (16)
N1	0.0300 (15)	0.059 (2)	0.0480 (19)	0.0030 (14)	−0.0002 (13)	−0.0056 (15)
C10	0.0390 (19)	0.054 (2)	0.070 (3)	0.0136 (17)	0.0019 (18)	0.0046 (19)
C7	0.0343 (18)	0.041 (2)	0.0307 (19)	0.0045 (15)	0.0025 (14)	0.0020 (15)
C1	0.051 (2)	0.057 (2)	0.050 (3)	0.0033 (19)	0.0052 (19)	0.0126 (19)
C3	0.051 (2)	0.042 (2)	0.046 (2)	0.0031 (18)	−0.0053 (18)	0.0098 (17)
C4	0.042 (2)	0.044 (2)	0.048 (2)	−0.0032 (17)	0.0117 (17)	−0.0020 (18)
C9	0.049 (2)	0.059 (3)	0.044 (2)	0.0245 (19)	−0.0006 (17)	−0.0078 (19)
C12	0.043 (2)	0.055 (2)	0.061 (3)	−0.0124 (18)	0.0046 (18)	−0.0097 (19)
C2	0.042 (2)	0.053 (3)	0.048 (2)	−0.0001 (18)	−0.0039 (17)	0.0102 (18)
C8	0.0300 (18)	0.072 (3)	0.054 (2)	0.0123 (19)	0.0010 (17)	−0.004 (2)
O5	0.0699 (18)	0.0627 (18)	0.0526 (18)	0.0014 (14)	−0.0212 (14)	0.0120 (14)
O3	0.0500 (16)	0.082 (2)	0.084 (2)	−0.0072 (14)	−0.0265 (15)	0.0217 (16)
O4	0.0776 (19)	0.0547 (18)	0.090 (2)	0.0011 (15)	0.0267 (16)	−0.0290 (16)
O2	0.083 (2)	0.0428 (18)	0.101 (2)	0.0090 (15)	−0.0070 (17)	−0.0054 (16)
O1	0.101 (2)	0.107 (3)	0.063 (2)	0.011 (2)	0.0383 (18)	0.0209 (18)

### Geometric parameters (Å, °)

**Table:**

Fe1—C1	1.803 (4)	C6—N2	1.314 (4)
Fe1—C2	1.784 (4)	C6—C7	1.387 (4)
Fe1—C3	1.780 (4)	N2—C9	1.356 (4)
Fe1—S1	2.2906 (9)	C5—O5	1.143 (4)
Fe1—S2	2.2893 (9)	N1—C7	1.320 (4)
Fe2—C4	1.761 (4)	N1—C8	1.360 (4)
Fe2—C5	1.771 (4)	C10—H10A	0.9600
Fe2—S1	2.2859 (9)	C10—H10B	0.9600
Fe2—S2	2.2783 (9)	C10—H10C	0.9600
Fe2—P1	2.2450 (9)	C1—O1	1.135 (4)
Fe2—Fe1	2.4970 (6)	C3—O3	1.132 (4)
S2—C6	1.787 (3)	C4—O4	1.148 (4)
S1—C7	1.782 (3)	C9—C8	1.352 (5)
P1—C11	1.809 (3)	C9—H9A	0.9300
P1—C10	1.815 (3)	C12—H12A	0.9600
P1—C12	1.815 (3)	C12—H12B	0.9600
C11—H11A	0.9600	C12—H12C	0.9600
C11—H11B	0.9600	C2—O2	1.139 (4)
C11—H11C	0.9600	C8—H8A	0.9300
			
C4—Fe2—C5	98.48 (16)	C10—P1—Fe2	116.66 (12)
C4—Fe2—P1	89.61 (11)	C12—P1—Fe2	117.62 (11)
C5—Fe2—P1	93.26 (10)	P1—C11—H11A	109.5
C4—Fe2—S2	153.63 (12)	P1—C11—H11B	109.5
C5—Fe2—S2	107.71 (11)	H11A—C11—H11B	109.5
P1—Fe2—S2	91.89 (3)	P1—C11—H11C	109.5
C4—Fe2—S1	91.40 (11)	H11A—C11—H11C	109.5
C5—Fe2—S1	99.45 (10)	H11B—C11—H11C	109.5
P1—Fe2—S1	166.96 (4)	N2—C6—C7	123.6 (3)
S2—Fe2—S1	81.51 (3)	N2—C6—S2	120.4 (2)
C4—Fe2—Fe1	97.83 (11)	C7—C6—S2	116.0 (2)
C5—Fe2—Fe1	151.59 (10)	C6—N2—C9	113.9 (3)
P1—Fe2—Fe1	109.96 (3)	O5—C5—Fe2	176.9 (3)
S2—Fe2—Fe1	57.07 (3)	C7—N1—C8	113.9 (3)
S1—Fe2—Fe1	57.02 (3)	P1—C10—H10A	109.5
C3—Fe1—C2	90.68 (15)	P1—C10—H10B	109.5
C3—Fe1—C1	100.37 (16)	H10A—C10—H10B	109.5
C2—Fe1—C1	98.81 (16)	P1—C10—H10C	109.5
C3—Fe1—S2	91.35 (11)	H10A—C10—H10C	109.5
C2—Fe1—S2	161.26 (11)	H10B—C10—H10C	109.5
C1—Fe1—S2	99.14 (12)	N1—C7—C6	122.7 (3)
C3—Fe1—S1	155.60 (12)	N1—C7—S1	120.3 (2)
C2—Fe1—S1	89.39 (11)	C6—C7—S1	117.0 (2)
C1—Fe1—S1	103.74 (12)	O1—C1—Fe1	179.1 (4)
S2—Fe1—S1	81.17 (3)	O3—C3—Fe1	178.1 (4)
C3—Fe1—Fe2	99.71 (11)	O4—C4—Fe2	179.7 (4)
C2—Fe1—Fe2	104.66 (11)	C8—C9—N2	122.8 (3)
C1—Fe1—Fe2	148.74 (11)	C8—C9—H9A	118.6
S2—Fe1—Fe2	56.65 (2)	N2—C9—H9A	118.6
S1—Fe1—Fe2	56.84 (2)	P1—C12—H12A	109.5
C6—S2—Fe2	101.60 (11)	P1—C12—H12B	109.5
C6—S2—Fe1	99.45 (11)	H12A—C12—H12B	109.5
Fe2—S2—Fe1	66.28 (3)	P1—C12—H12C	109.5
C7—S1—Fe2	100.61 (10)	H12A—C12—H12C	109.5
C7—S1—Fe1	99.80 (11)	H12B—C12—H12C	109.5
Fe2—S1—Fe1	66.13 (3)	O2—C2—Fe1	178.7 (3)
C11—P1—C10	101.87 (16)	C9—C8—N1	123.1 (3)
C11—P1—C12	102.21 (17)	C9—C8—H8A	118.5
C10—P1—C12	102.53 (16)	N1—C8—H8A	118.5
C11—P1—Fe2	113.71 (11)		
			
C4—Fe2—Fe1—C3	86.09 (15)	C5—Fe2—S1—Fe1	−162.74 (11)
C5—Fe2—Fe1—C3	−149.4 (2)	P1—Fe2—S1—Fe1	4.13 (16)
P1—Fe2—Fe1—C3	−6.35 (12)	S2—Fe2—S1—Fe1	−56.06 (3)
S2—Fe2—Fe1—C3	−85.19 (12)	C3—Fe1—S1—C7	−115.1 (3)
S1—Fe2—Fe1—C3	172.66 (12)	C2—Fe1—S1—C7	154.63 (15)
C4—Fe2—Fe1—C2	−7.18 (15)	C1—Fe1—S1—C7	55.71 (16)
C5—Fe2—Fe1—C2	117.4 (2)	S2—Fe1—S1—C7	−41.61 (10)
P1—Fe2—Fe1—C2	−99.62 (11)	Fe2—Fe1—S1—C7	−97.35 (10)
S2—Fe2—Fe1—C2	−178.46 (11)	C3—Fe1—S1—Fe2	−17.7 (3)
S1—Fe2—Fe1—C2	79.39 (11)	C2—Fe1—S1—Fe2	−108.02 (11)
C4—Fe2—Fe1—C1	−144.6 (3)	C1—Fe1—S1—Fe2	153.05 (12)
C5—Fe2—Fe1—C1	−20.0 (3)	S2—Fe1—S1—Fe2	55.73 (3)
P1—Fe2—Fe1—C1	123.0 (2)	C4—Fe2—P1—C11	85.98 (17)
S2—Fe2—Fe1—C1	44.1 (2)	C5—Fe2—P1—C11	−12.49 (17)
S1—Fe2—Fe1—C1	−58.0 (2)	S2—Fe2—P1—C11	−120.35 (13)
C4—Fe2—Fe1—S2	171.28 (11)	S1—Fe2—P1—C11	−179.51 (18)
C5—Fe2—Fe1—S2	−64.2 (2)	Fe1—Fe2—P1—C11	−175.83 (13)
P1—Fe2—Fe1—S2	78.84 (4)	C4—Fe2—P1—C10	−32.18 (19)
S1—Fe2—Fe1—S2	−102.15 (3)	C5—Fe2—P1—C10	−130.65 (18)
C4—Fe2—Fe1—S1	−86.57 (11)	S2—Fe2—P1—C10	121.49 (14)
C5—Fe2—Fe1—S1	38.0 (2)	S1—Fe2—P1—C10	62.3 (2)
P1—Fe2—Fe1—S1	−179.01 (4)	Fe1—Fe2—P1—C10	66.01 (15)
S2—Fe2—Fe1—S1	102.15 (3)	C4—Fe2—P1—C12	−154.65 (18)
C4—Fe2—S2—C6	−115.1 (3)	C5—Fe2—P1—C12	106.88 (17)
C5—Fe2—S2—C6	57.96 (15)	S2—Fe2—P1—C12	−0.98 (14)
P1—Fe2—S2—C6	151.99 (11)	S1—Fe2—P1—C12	−60.1 (2)
S1—Fe2—S2—C6	−39.31 (11)	Fe1—Fe2—P1—C12	−56.46 (14)
Fe1—Fe2—S2—C6	−95.33 (11)	Fe2—S2—C6—N2	−147.9 (2)
C4—Fe2—S2—Fe1	−19.8 (2)	Fe1—S2—C6—N2	144.6 (2)
C5—Fe2—S2—Fe1	153.29 (10)	Fe2—S2—C6—C7	30.9 (2)
P1—Fe2—S2—Fe1	−112.68 (3)	Fe1—S2—C6—C7	−36.6 (2)
S1—Fe2—S2—Fe1	56.02 (3)	C7—C6—N2—C9	−0.5 (4)
C3—Fe1—S2—C6	−160.66 (15)	S2—C6—N2—C9	178.2 (2)
C2—Fe1—S2—C6	103.2 (4)	C8—N1—C7—C6	−0.5 (5)
C1—Fe1—S2—C6	−59.94 (16)	C8—N1—C7—S1	179.2 (2)
S1—Fe1—S2—C6	42.68 (11)	N2—C6—C7—N1	1.1 (5)
Fe2—Fe1—S2—C6	98.60 (11)	S2—C6—C7—N1	−177.7 (2)
C3—Fe1—S2—Fe2	100.74 (12)	N2—C6—C7—S1	−178.7 (2)
C2—Fe1—S2—Fe2	4.6 (3)	S2—C6—C7—S1	2.6 (3)
C1—Fe1—S2—Fe2	−158.53 (12)	Fe2—S1—C7—N1	145.7 (2)
S1—Fe1—S2—Fe2	−55.92 (3)	Fe1—S1—C7—N1	−146.9 (2)
C4—Fe2—S1—C7	−165.46 (16)	Fe2—S1—C7—C6	−34.5 (2)
C5—Fe2—S1—C7	−66.63 (15)	Fe1—S1—C7—C6	32.8 (2)
P1—Fe2—S1—C7	100.24 (18)	C6—N2—C9—C8	−0.5 (5)
S2—Fe2—S1—C7	40.04 (11)	N2—C9—C8—N1	1.1 (6)
Fe1—Fe2—S1—C7	96.11 (11)	C7—N1—C8—C9	−0.5 (5)
C4—Fe2—S1—Fe1	98.43 (12)		
